# Short telomeres in alveolar type II cells associate with lung fibrosis in post COVID-19 patients with cancer

**DOI:** 10.18632/aging.204755

**Published:** 2023-06-07

**Authors:** Paula Martínez, Raúl Sánchez-Vazquez, Arpita Saha, Maria S. Rodriguez-Duque, Sara Naranjo-Gonzalo, Joy S. Osorio-Chavez, Ana V. Villar-Ramos, Maria A. Blasco

**Affiliations:** 1 Telomeres and Telomerase Group, Molecular Oncology Program, Spanish National Cancer Centre (CNIO), Madrid E-28029, Spain; 2 Servicio de Anatomía Patológica, Hospital Universitario Marqués de Valdecilla, Santander 39008, Spain; 3 Servicio de Cirugía Torácica, Hospital Universitario Marqués de Valdecilla, Santander 39008, Spain; 4 Servicio de Neumología Hospital Universitario Marqués de Valdecilla, Santander E-39008, Spain; 5 Institute of Biomedicine and Biotechnology of Cantabria (IBBTEC), Cantabria, Santander E-39011, Spain; 6 Instituto de Investigación Marqués de Valdecilla (IDIVAL), Santander E-39011, Spain; 7 Departamento de Fisiología y Farmacología, Universidad de Cantabria, Santander E-39011, Spain

**Keywords:** ATII cells, lung fibrosis, telomeres, COVID-19, SARS-CoV2

## Abstract

The severe acute respiratory syndrome coronavirus 2 (SARS-CoV-2) is responsible for the coronavirus disease 2019 (COVID-19) pandemic. The severity of COVID-19 increases with each decade of life, a phenomenon that suggest that organismal aging contributes to the fatality of the disease. In this regard, we and others have previously shown that COVID-19 severity correlates with shorter telomeres, a molecular determinant of aging, in patient’s leukocytes. Lung injury is a predominant feature of acute SARS-CoV-2 infection that can further progress to lung fibrosis in post-COVID-19 patients. Short or dysfunctional telomeres in Alveolar type II (ATII) cells are sufficient to induce pulmonary fibrosis in mouse and humans. Here, we analyze telomere length and the histopathology of lung biopsies from a cohort of alive post-COVID-19 patients and a cohort of age-matched controls with lung cancer. We found loss of ATII cellularity and shorter telomeres in ATII cells concomitant with a marked increase in fibrotic lung parenchyma remodeling in post- COVID-19 patients compared to controls. These findings reveal a link between presence of short telomeres in ATII cells and long-term lung fibrosis sequel in Post-COVID-19 patients.

## INTRODUCTION

The lung parenchyma or alveolar region of the lung is where the gas exchange takes place and corresponds to 80-90% of the total lung volume. The remaining non-parenchyma consists of the conducting airways, trachea, bronchi and bronchioles, as well as blood vessels. The alveolar epithelium is composed by alveolar type I (ATI) and alveolar type II (ATII) cells, both with distinct functional specialization and structural differentiation [1]. ATI cells comprise the major gas exchange surface of the alveolus and are key for the maintenance of the permeability barrier of the alveolar membrane. ATII are the progenitor cells for ATI and also responsible for surfactant production and homeostasis [2]. ATI cells are more sensitive to injuries that ATII [3]. Once ATI cells are damaged, adjacent ATII are stimulated to proliferate and transdifferentiate into ATI. ATII cells with self-renewal capacity function as progenitors for the lung parenchyma restoring the epithelium after lung injury [1]. Thus, it is conceivable that cumulative damage to ATI or ATII cells could be at the origin of exhaustion of the regenerative potential of lungs and lung degenerative diseases.

Telomeres are specialized structures at the chromosome ends, which are essential for chromosome-end protection and genomic stability. Vertebrate telomeres consist of tandem repeats of the TTAGGG DNA sequence bound by a six-protein complex known as shelterin, which prevents chromosome end-to-end fusions, telomere fragility, and the induction of a persistent DNA damage response [4, 5]. As cells divide and DNA has to be replicated, telomeres become progressively shorter owing to the so-called “end replication problem” [6, 7]. Thus, telomere shortening occurs associated with increasing age in humans [8], mice [9] and other species, and the rate of telomere shortening has been shown to correlate with species lifespan [10]. When telomeres become critically short this results in loss of telomere protection and telomere damage, leading to activation of a persistent DNA damage response and loss of cellular viability by induction of apoptosis and/or senescence [4, 5].

Idiopathic pulmonary fibrosis (IPF) is a rare lung disease characterized by progressive loss of functional lung cells and fibrosis of the lung parenchyma. IPF can be both a sporadic and a familial disease. The familial cases are linked to mutations in surfactant related genes or in telomere maintenance genes [11–13]. Sporadic cases of IPF, not associated with telomere maintenance genes mutations, also show shorter telomeres in blood and in ATII cells compared to age-matched controls, with 25% of the patients showing telomeres as short as the telomerase mutation carriers [14, 15].

Of importance, induction of telomere dysfunction specifically in alveolar type II (ATII) cells, and not in other cell types including fibroblast, basal cells or Clara cells, is sufficient to induce progressive and lethal pulmonary fibrosis in lung parenchyma in mice, which is concomitant with induction of telomeric DNA damage, cell death and cellular senescence [16–20]. Indeed, in fibrotic human lungs as well as in mouse models for lung fibrosis, senescent ATII are observed [1, 2, 17, 21, 22], in line with the notion that telomere shortening or telomere dysfunction could be at the origin of this degenerative lung disease.

The severe acute respiratory syndrome coronavirus 2 (SARS-CoV-2) is responsible for the coronavirus disease 2019 (COVID-19) pandemic. COVID-19 severity has been associated to increasing age of the infected patients, suggesting a component of aging in the disease [23, 24]. In this regard, we and others have shown that the severity of the COVID-19 disease is associated with higher abundance of shorter telomere length in patient’s leukocytes [23, 25, 26], raising the hypothesis that short telomeres maybe contributing to the COVID-19 disease.

Of interest, the SARS-CoV-2 virus uses the angiotensin-converting enzyme 2 (ACE2) as the receptor for cellular entry [27, 28]. In normal human lungs, Type II alveolar (ATII) epithelial cells constitute the majority of ACE2-expressing lung cells (83%) although only 1.4% of ATII cells express ACE2 indicating that ACE2 expression does take place in a special small population of ATII cells. The remaining 17% of ACE2-expressing cells in the lung include type I alveolar (ATI), airway epithelial cells, fibroblast, macrophages and endothelial cells [29]. The fact that SARS-CoV-2 targets ATII cells in the lung explains the severe alveolar damage in severe COVID-19 patients [29]. Of interest, lung injury is a predominant feature of acute SARS-CoV-2 infection. The abnormal imaging pattern of COVID-19 patients with severe pneumonia suggests that these patients are likely at an increased risk of progression to post-COVID-19 development of lung fibrosis with permanent functional impairment. Indeed, post-COVID-19 pulmonary fibrosis is being considered as a major sequelae of the pandemic [30].

Here, we set to address whether short telomeres in the lungs of post- COVID-19 patients could be at the origin of virus-induced pulmonary fibrosis. To this end, we performed histopathological analysis of lung biopsies from a cohort of 19 alive post-COVID-19 patients and 79 age-matched controls, and found marked fibrotic lung parenchyma remodeling with fibroblast proliferation, airspace destruction and reduced ATII abundance in post-COVID-19 patients compared to controls. Most relevant, post-COVID-19 patients presented with shorter telomeres in ATII cells compared to age-matched controls. These findings suggest a role for short telomeres in the origin of severe COVID-19 sequelae, such as pulmonary fibrosis.

## RESULTS

### Cohort of COVID-19 patients and age-matched controls

In order to study a potential role of short telomeres in the origin of the post-viral lung fibrosis sequel presented by a percentage of COVID-19 patients, paraffin-embedded lung samples from post-COVID-19 and COVID-19-free patients were obtained from the University Hospital of Marques de Valdecilla in Santander, Spain. In all cases, lung samples corresponded to biopsies from non-tumoral areas of the lungs from lung cancer patients. The samples used for the analysis were confirmed to correspond to normal, non-tumoral tissue by using pathological analysis. Cancer patients were used owing to the fact that normal lung biopsies were readily available. A total of 35 females and 63 males of ages ranging from 42 to 84 years old were included in the study (Table 1). Nineteen patients, 5 females and 14 males, passed the COVID-19 disease previous to lung surgery, while the rest of the patients never had the COVID-19 disease. The clinical and demographic characteristics of patients under the study are summarized in Table 2. Of note, histopathological analysis of lung samples post-surgery showed that 59% of COVID-19 patients (11 out of 19) and 19% of control patients (15 out of 79) presented lung fibrosis (Tables 1, 2). It should be pointed out that none of the COVID-19 patients had fibrosis prior to SARS CoV-2 infection based on either chest radiographs or CT scans performed before infection. None of the COVID-19 patients had CT lung pannalization characteristic of the usual interstitial pneumonia. Of note, echocardiographic findings are suggestive of pulmonary hypertension in only one patient from control group.

**Table 1:** Control and COVID-19 patients under study.

	**Sex**	**Age (years)**	**Biopsy**	**Fibrosis**
**Controls**				
Control 1	Male	45	B20-4315	No
Control 2	Female	59	B20-16593	No
Control 3	Female	83	B20-20700	No
Control 4	Male	78	B21-1572	No
Control 5	Male	65	B21-2679	No
Control 6	Male	84	B21-2688	No
Control 7	Female	80	B21-470	No
Control 8	Female	62	B21-571	No
Control 9	Female	66	B21-613	No
Control 10	Female	63	B21-1042	No
Control 11	Male	54	B21-1132	No
Control 12	Female	71	B21-1599	No
Control 13	Male	60	B21-2199	Yes
Control 14	Male	78	B21-2358	Yes
Control 15	Male	66	B21-2679	No
Control 16	Female	69	B21-2739	No
Control 17	Male	68	B21-4443	No
Control 18	Male	77	B21-7920	No
Control 19	Male	73	B21-7869	No
Control 20	Male	73	B21-8659	No
Control 21	Male	69	B21-9201	No
Control 22	Female	76	B21-9647	No
Control 23	Male	64	B21-11060	No
Control 24	Male	64	B21-11606	Yes
Control 25	Female	70	B21-12024	No
Control 26	Male	68	B21-12168	Yes
Control 27	Male	72	B21-12186	Yes
Control 28	Male	73	B21-12752	No
Control 29	Male	66	B21-13359	No
Control 30	Female	66	B21-14681	No
Control 31	Female	62	B21-14588	No
Control 32	Male	58	B21-15032	No
Control 33	Female	42	B21-15986	No
Control 34	Male	59	B21- 16407	No
Control 35	Female	84	B21- 16496	No
Control 36	Male	60	B21- 18388	No
Control 37	Male	47	B21- 18468	No
Control 38	Female	50	B21- 18433	No
Control 39	Female	68	B21- 18891	No
Control 40	Female	63	B21-19598	No
Control 41	Male	66	B21-20680	No
Control 42	Male	77	B21-20835	No
Control 43	Male	72	B21-21282	No
Control 44	Male	66	B21-22922	No
Control 45	Male	66	B21-23007	No
Control 46	Male	80	B21-23575	No
Control 47	Male	70	B21-23641	No
Control 48	Female	61	B21-24222	No
Control 49	Female	72	B21-24182	No
Control 50	Male	74	B21-25026	Yes
Control 51	Female	66	B21-25449	Yes
Control 52	Male	79	B21-26566	No
Control 53	Male	72	B21-26640	No
Control 54	Female	74	B21-27377	No
Control 55	Male	73	B21-27275	No
Control 56	Male	77	B21-27490	No
Control 57	Male	68	B21-27550	No
Control 58	Female	53	B22-398	No
Control 59	Male	60	B22-1485	No
Control 60	Male	79	B22-1607	No
Control 61	Female	74	B22-2114	No
Control 62	Male	57	B22-2267	Yes
Control 63	Female	68	B22-3903	No
Control 64	Male	71	B22-4103	No
Control 65	Female	73	B22-5408	Yes
Control 66	Male	72	B22-5333	Yes
Control 67	Female	58	B22-5843	No
Control 68	Male	74	B22-5932	No
Control 69	Male	71	B22-6089	No
Control 70	Male	69	B22-6592	Yes
Control 71	Female	58	B22-7124	Yes
Control 72	Male	71	B22-7226	No
Control 73	Male	66	B22-8331	Yes
Control 74	Male	79	B22-8447	Yes
Control 75	Female	63	B22-8816	No
Control 76	Male	70	B22-9475	No
Control 77	Female	83	B22-9599	No
Control 78	Female	63	B22-9988	No
Control 79	Male	82	B22-10535	Yes
**Covids**				
Covid 1	Female	82	B20-15688	Yes
Covid 2	Male	67	B20-9722	Yes
Covid 3	Female	69	B20-8325	Yes
Covid 4	Male	66	B21-1539	No
Covid 5	Male	73	B21-6981	No
Covid 6	Male	77	B21-10260	Yes
Covid 7	Male	65	B21-11564	Yes
Covid 8	Male	72	A21-6	Yes
Covid 9	Male	66	B21-5757	No
Covid 10	Male	79	B21-13286	Yes
Covid 11	Female	48	B21- 18230	No
Covid 12	Male	79	B21-25575	Yes
Covid 13	Male	62	B22-508	No
Covid 14	Male	64	B22-1037	Yes
Covid 15	Female	64	B22-4011	No
Covid 16	Male	76	B22-6717	Yes
Covid 17	Male	64	B22-8708	No
Covid 18	Female	84	B22-9363	Yes
Covid 19	Male	72	B22-10050	No

Results from histopathological diagnose of lung fibrosis post-surgery are indicated.

**Table 2:** Clinical data of patients.

**Variable**	**COVID19**	**Surgical controls**
Number	19	79
Gender (F/M)	5/14	35/64
Age (years)	70±9	68±9
Current smoking (yes/no)	3/16	23/56
COVID19 mild/severe	11/8	-
Pre-COVID19 lung fibrosis diagnosed by molecular imaging (yes/no)	0/19	-
Post-surgery lung fibrosis diagnosed by histopathology (yes/no)	11/8	15/64
Pulmonary Hypertension (yes/no)	0/19	1/78
Concomitant diseases (C/R/D)	6/3/2	21/24/11
CT scan with ground opacity (yes/no)	7/12	-
CT scan pulmonary effusion	13/6	-
Pulmonary function test (positive/negative)	13/6	-
Respiratory bronchiolitis	5/14	28/51
Treatment with immunosuppressants or immunomodulators (yes/no)	5/14	6/73

Patient demographic and clinical characteristics are presented as mean ± standard deviation. F, female; M, male; C, cardiovascular; R, Respiratory; D, diabetes, - not performed.

### Progressive telomere shortening with age in lung parenchyma

**Figure 1:**
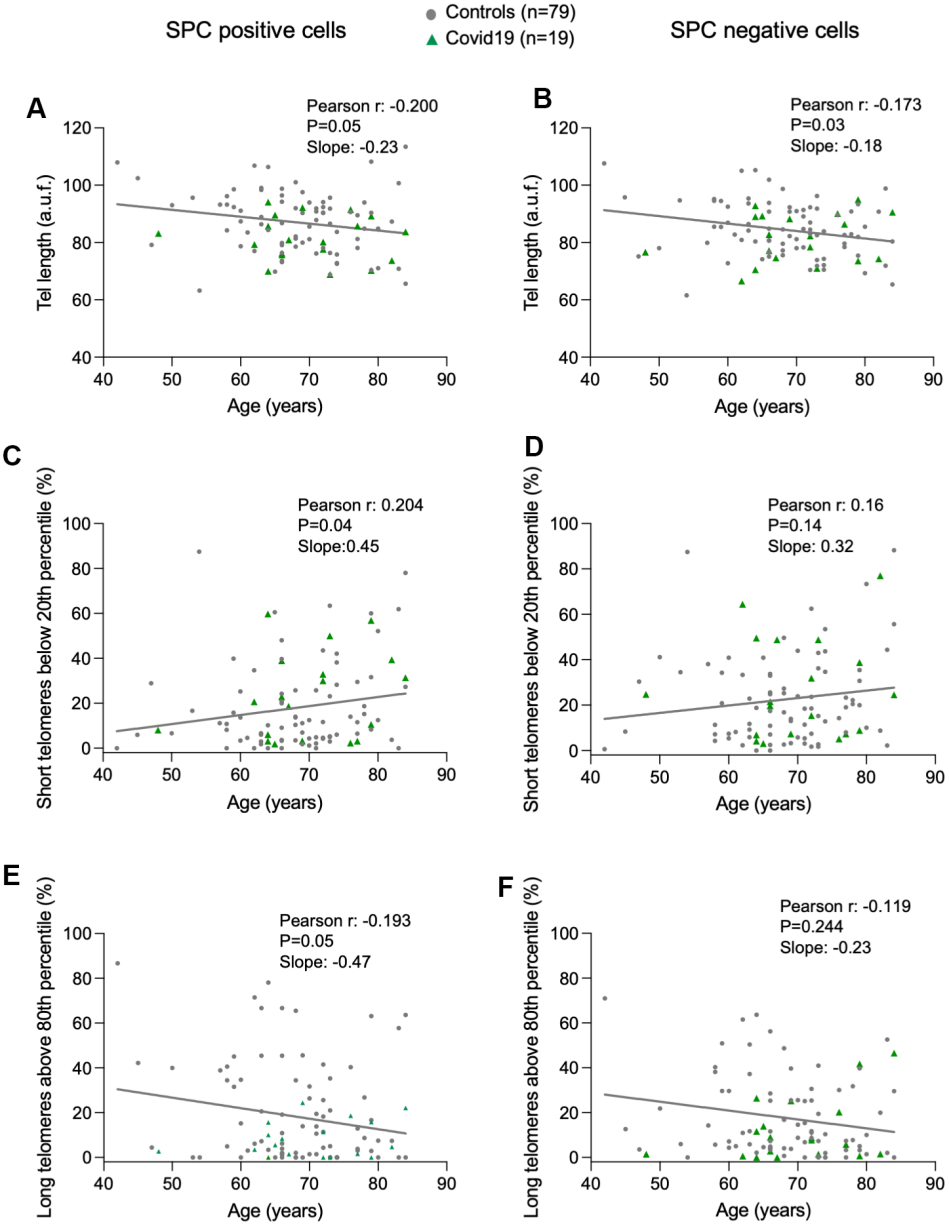
**Progressive telomere shortening with age in lung cells.** (**A**–**F**) Linear regression and Pearson correlation analyses between telomere intensity in pro-SPC positive (**A**) and pro-SPC negative cells (**B**), between percentage of short telomeres in pro-SPC positive (**C**) and pro-SPC negative cells (**D**), and between percentage of long telomeres in pro-SPC positive (**E**) and pro-SPC negative cells (**F**) and age in lung section from patients under study including control and COVID-19 samples. The Pearson r coefficient and the P value are indicated.

To address the potential role of telomere length in COVID-19 associated lung pathologies, we analyzed telomere length in a single-cell manner in lung cells from the lung parenchyma by using Quantitative telomere Fluorescence *In Situ* Hybridization (Q-FISH), a technique that allows quantification of individual telomere fluorescence spots per interphasic nuclei in tissue sections, which in turn serve to quantify telomere length [31]. To distinguish between ATII and non-ATII cells in the lung parenchyma, we combined the Q-FISH technique with an immunofluorescence staining using specific antibody against prosurfactant protein C (pro-SPC), a bona fide marker of ATII cells. First, we represented the distribution of the mean telomere spot intensity per nucleus in ATII and non-ATII cells from each control and COVID-19 samples (Supplementary Figure 1). Linear regression analysis between average of mean telomere spot intensity per nucleus and patient’s age, revealed a significant telomere shortening with increasing age in both ATII and non-ATII lung cells, something that has been described for other cell types previously but not for lung cell populations in humans [32–35] (Figure 1A, 1B). In agreement with telomere shortening with increasing patient age in lung cells, we also observed statistically significant increase in the abundance of short telomeres (ie, telomere fluorescence below 20^th^ percentile of all telomeres in control samples) and a significant decrease in the abundance of long telomeres (ie, telomere fluorescence above 80^th^ percentile of all telomeres in control samples) only in ATII cells but not in non-ATII cells (Figure 1C–1F). This observation could be explained by the fact that the progenitor nature of ATII cells could lead to a higher proliferative history compared to non-ATII cells within the lung parenchyma and a consequently higher rate of telomere shortening associated to cell division with age. Consistent with this notion, we found a higher rate of decrease of telomere length, a higher rate of increase in the abundance of short telomeres, as well as a higher rate of decrease in the abundance of long telomeres with age in ATII cells compared with non-ATII cells in the linear regression models (Figure 1A–1F).

### COVID-19 patients present shorter telomeres in ATII cells than age-matched controls

**Figure 2:**
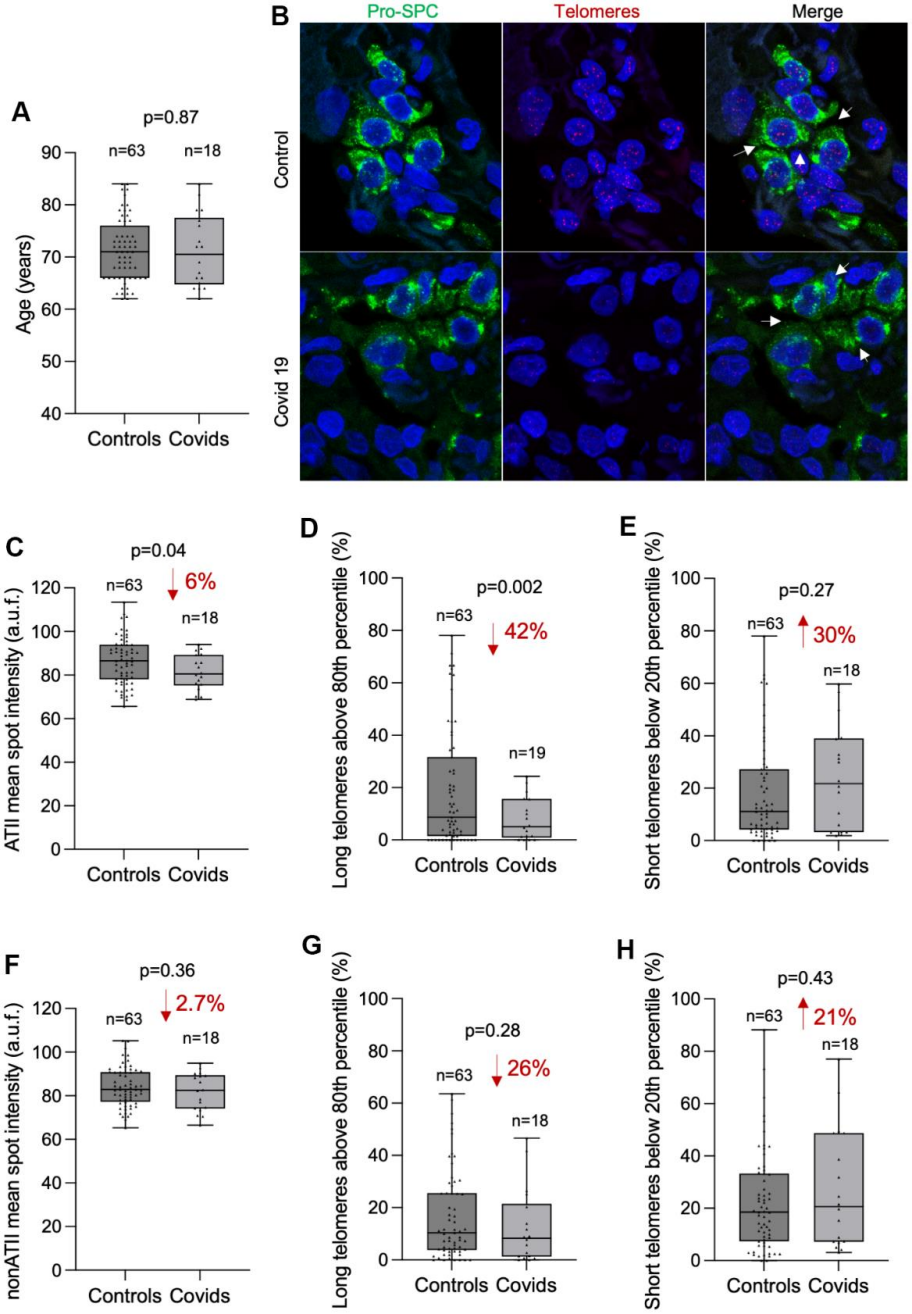
**COVID-19 patients present shorter telomeres in the alveolar type II cells than controls.** (**A**) Box and Whisker plot representation of control and COVID-19 patients’ age within 62 and 84 years old. Differences in age distributions between both control and COVID-19 sample groups was analyzed by Kolmogorov-Smirnov test. The p-value is indicated. (**B**) Representative images of telomere quantitative fluorescence *in situ* hybridization (q-FISH) combined with immunofluorescence against Pro-SPC in a control and a COVID-19 lung samples. (**C**–**H**) Box and Whisker plot representation of mean telomeric spot intensity per nucleus (**C**, **F**), percentage of long telomeres above 80^th^ percentile (**D**, **G**) and percentage of short telomeres below 20^th^ percentile (**E**, **H**) in alveolar type II (ATII) (**C**–**E**) and in non-ATII cells (**F**–**H**) in lung sections from control and COVID-19 patients within 62 and 84 year-old age interval. Statistical significance in (**C**–**H**) was assessed using unpaired Student’s t test with Welch’s correction and the p-value is indicated. The percent increase or decrease between control and COVID-19 samples are indicated in each plot.

In order to assess the potential correlation between telomere length in lung tissue and COVID-19 disease, we compared the average mean telomere intensity per nucleus of COVID-19 patients with that of controls both in ATII and non-ATII cells within the lung parenchyma (Figure 2A–2H). We performed the analysis in age-matched individuals within an age interval from 62 to 84 year old in which most of COVID-19 patients are found (18 out of 19 COVID-19 samples). First, we used the Kolmogorov-Smirnov (KS) test to assess whether the cumulative frequency distribution of patient’s age from control and COVID-19 groups were different. The KS test revealed similar age distribution in control and COVID-19 patients ruling out bias in the potential differences in telomere length due to different ages between both groups (Figure 2A).

Then, we compared the mean telomere intensity per nucleus in ATII cells in both control and COVID-19 patients and found a significant 6% lower mean telomere fluorescence intensity in ATII cells from COVID-19 patients compared to those from controls (Figure 2C). In accordance with this, we also found a significant 42% decrease in the abundance of long telomeres above the 80^th^ percentile in the telomeres of control samples (Figure 2D). We also found a 30% increase in the abundance of short telomeres below 20^th^ percentile of control samples although this difference did not reach statistical significance (Figure 2E). Interestingly, although we also observe a similar trend in telomere length, long and short telomere abundance in non-ATII cells, none of these comparisons reached statistical significance (Figure 2F–2H). These observations support the fact that SARS-Cov2 mainly infects ATII cells, and thus can lead to increased turn-over of the remaining progenitor ATII cells in an attempt to regenerate the COVID-19 induced lung damage, which in turn accelerates telomere shortening in these cells. Short/dysfunctional telomeres in ATII cells impair lung damage repair and are sufficient to trigger interstitial pulmonary fibrosis in mouse models [17].

### COVID-19 patients present lung fibrosis

**Figure 3:**
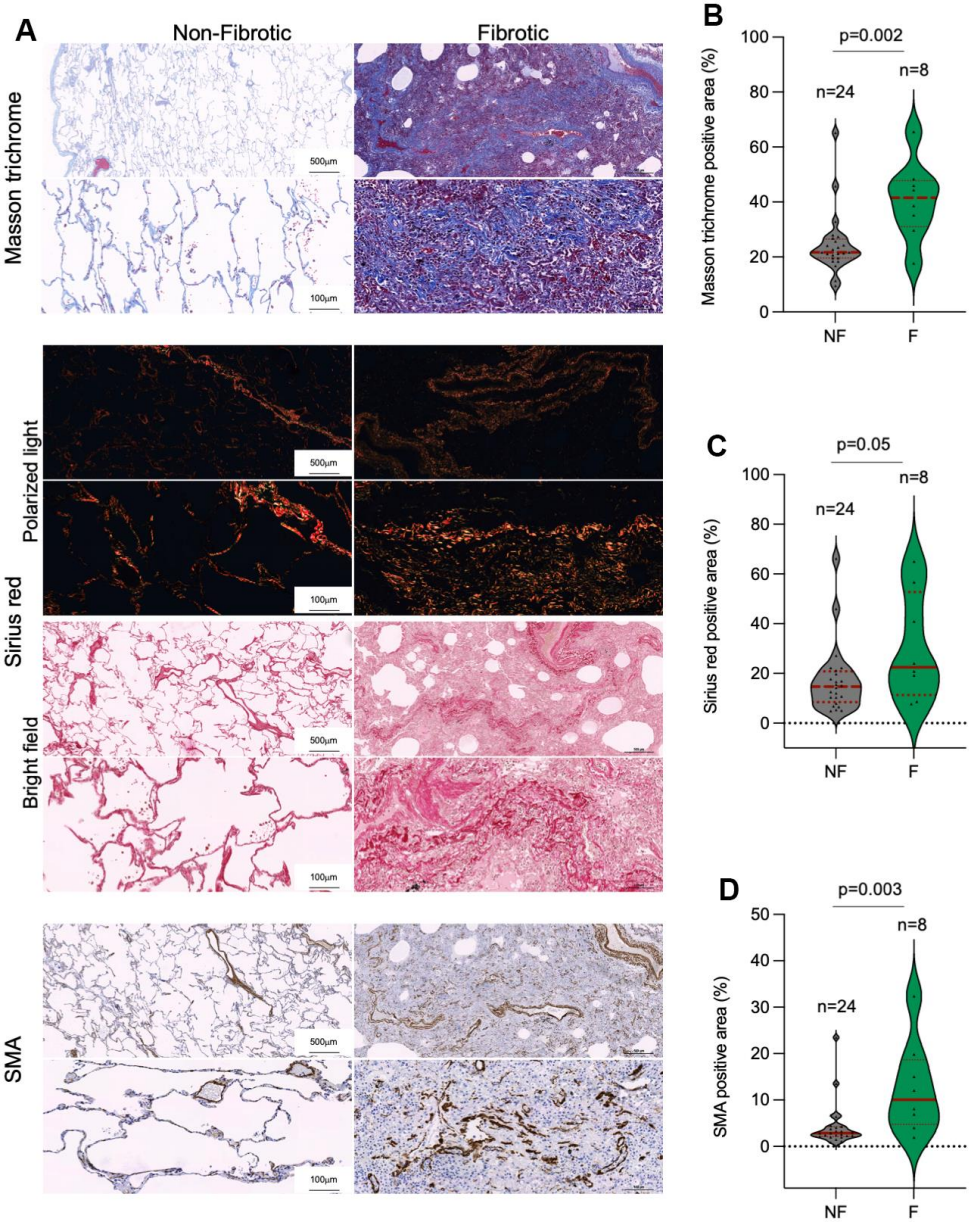
**Collagen depots in patients diagnosed with lung fibrosis post-surgery.** (**A**) Representative images of Masson trichrome, Sirius red (polarized light and bright field) and smooth muscle actine (SMA) staining in non-fibrotic and fibrotic lungs. (**B**–**D**) Quantification of Masson trichrome (**B**), Sirius red (**C**) and SMA (**D**) positive stained lung area in non-fibrotic (NF) and fibrotic (F) lung samples. The samples analyzed correspond to Control 1-23 and COVID-19 1-9 (Table 1). Statistical significance was assessed using Student’s t test and the p-value is indicated.

In line with the above, a potential outcome of SARS-CoV-2 infection is the induction of fibrosis-like phenotypes in the lung, suggesting that the viral infection maybe exhausting the regenerative potential of lung tissue [36–39]. Indeed, histopathological analysis of lung samples post-surgery showed that the incidence of lung fibrosis was 3-fold higher among COVID-19 than in control patients, 59% and 19%, respectively (Table 2). We further analyzed the lung fibrotic pathologies in a subset of our patient cohorts by performing Masson trichrome and Sirius red staining for histological evaluation of collagen fibers and by immunohistochemical staining of α-smooth muscle actin (SMA), a marker of activated fibroblasts and myofibroblasts that secrete extracellular components required for wound repair [40]. Quantitative analysis of Masson trichrome, Sirius red and SMA revealed that histopathologically diagnosed with pulmonary fibrosis post-surgery in control and COVID-19 patient cohorts present significant higher collagen depots and increased presence of activated fibroblasts and/or myofibroblasts as compared to control lungs (Figure 3A–3D).

**Figure 4:**
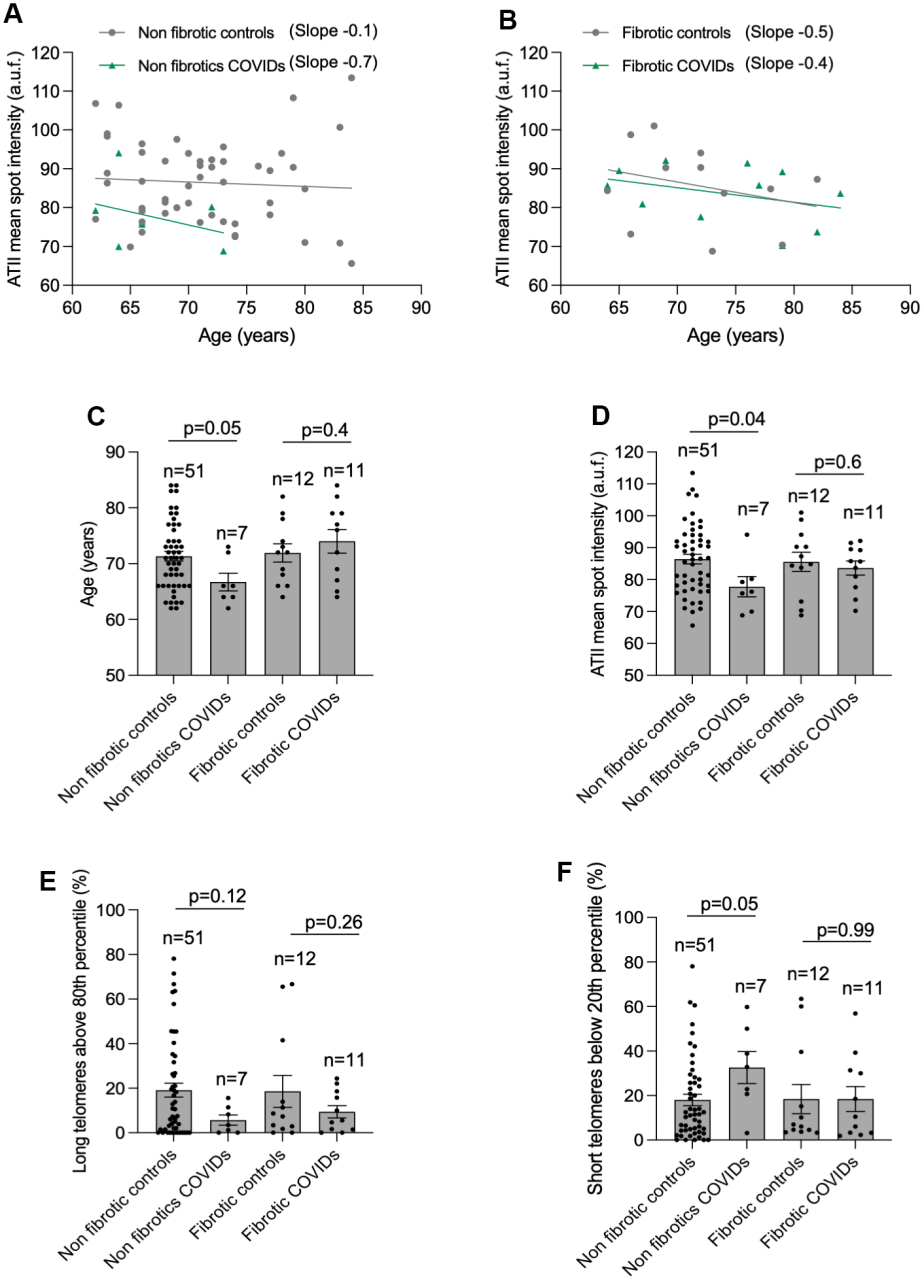
**Higher rate of telomere shortening with age in COVID-19 patients.** (**A**, **B**) Linear regression analyses between telomere intensity in pro-SPC positive cells and age in lung section from control and COVID-19 patients not presenting lung fibrosis (**A**) or being histopathologically diagnosed with lung fibrosis (**B**) post-surgery. The slope of the linear regression is shown. (**C**) Mean age of control and COVID-19 patients within 62 and 84 year-old interval presenting or not lung fibrosis post-surgery. (**D**–**F**) Mean telomeric spot intensity per nucleus (**D**), percentage of long telomeres above 80^th^ percentile (**E**) and percentage of short telomeres below 20^th^ percentile (**F**) in alveolar type II in lung sections from control and COVID-19 patients within 62 and 84 year-old age interval. Statistical significance in (**C**–**F**) was assessed using unpaired Student’s t test and the p-value is indicated.

We next compared telomere length and the percentage of long and short telomeres in ATII cells in histopathological diagnosed fibrotic and no-fibrotic patients within the age range of 62- to 84-year-old (Table 1 and Figure 4). The results clearly showed that non-fibrotic COVID-19 patients showed a 7-fold higher rate of telomere shortening than their age-matched non-fibrotic controls (Figure 4A). In contrast, the rate of telomere shortening was similar when comparing fibrotic controls with fibrotic COVID-19 patients (Figure 4B). Non-fibrotic COVID-19 patients present shorter mean telomere length, increased percentage of short telomeres and lower percentage of long telomeres in ATII cells as compared to age-matched non-fibrotic control patients (Figure 4C–4G), supporting the notion that short telomeres are a risk factor for SARS-CoV2 infection [23, 25, 26]. It should be pointed out that the difference in short telomeres between non-fibrotic COVID-19 and control patients is even observed in a COVID-cohort younger than its correspondent control-cohort (mean age of 66 and 71 in COVID-19 and controls, respectively) (Figure 4C). No significant differences were detected in mean telomere length and the percentage of long/short telomeres between fibrotic control and fibrotic COVID-19 cohorts (Figure 4D–4F), in agreement with the notion that short telomeres associate with pulmonary fibrosis [14, 15].

### Decreased number of alveolar type II cells in the lungs of COVID-19 patients

**Figure 5:**
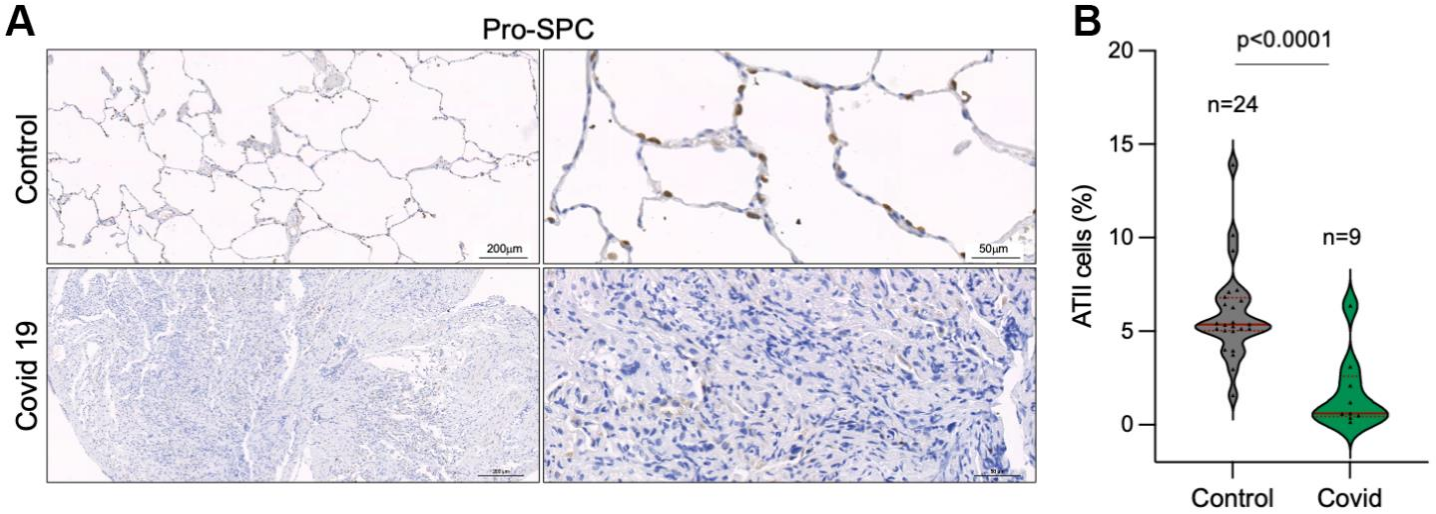
**Decreased number of alveolar type II cells in the lungs of COVID-19 patients.** (**A**, **B**) Representative images of prosurfactant protein C (pro-SPC) immunostaining, a marker of alveolar type II cells (ATII) (**A**) and quantification of pro-SpC positive cells in the lungs of control and COVID-19 patients (**B**) Statistical significance was assessed using Student’s t test and the p-value is indicated.

As the SARS-CoV-2 virus in known to infect mainly ATII cells [29, 41], we set to analyze the percentage of ATII cells in control and COVID-19 samples by immune staining of Pro-surfactant protein C (Pro-SPC) as a specific marker of ATII cells. The results clearly show a significant lower number of ATII cells in COVID-19 patients compared to control lung samples (Figure 5A, 5B). Since ATII cells constitute the alveolar regenerative stem cells, this observation supports the notion that SARS-CoV-2 infection induces a regenerative/proliferative response that leads to further telomere shortening in ATII cells that may contribute to ATII exhaustion.

## DISCUSSION

There is growing evidence of histopathological changes consistent with lung fibrosis in autopsied individuals infected with SARS-CoV-2 who died with COVID-19 [42–44]. However, lung samples with histopathological information in post-COVID-19 survivors is highly limited. There are few case reports of post-COVID-19 patients that died from diseases other than acute COVID-19 whose lung histopathology examination revealed extensive lung fibrotic changes with interstitial pattern (https://pesquisa.bvsalud.org/global-literature-on-novel-coronavirus-2019-ncov/resource/pt/covidwho-1857241). In particular, there is one case report of a post-COVID-19 patient that died of lung failure as the consequence of widespread pulmonary fibrosis without a previous history of pulmonary illness [45]. These findings of histologically-proven lung fibrosis long after SARS-CoV2 infection resolution provide strong evidence of long-lasting lung fibrosis sequel in post-COVID-19 patients.

Here, we analyze lung biopsies from a cohort of alive post-COVID-19 patients and a cohort of patients who had not passed the disease, which we used as controls. We found significantly higher incidence of fibrotic lung parenchyma remodeling with fibroblast proliferation and myofibroblast differentiation, airspace obliteration and reduced ATII abundance in post-COVID-19 patients compared to controls. These findings suggest that the previous SARS-CoV-2 infection was causative of PF development. We also found that post-COVID-19 patients as well as control patients that present lung fibrosis post-surgery show significantly shorter telomeres than age matched controls in the lung parenchyma, specifically in ATII cells. These observations might reflect that the SARS-CoV-2 infection of ATII cells induces telomere shortening as a consequence of enhanced proliferative response to regenerate the alveolar damage leading to exhaustion of the regenerative potential of the lung tissue. In this regard, telomere exhaustion in ATII cells will be more likely to happen in older patients, which already have shorter telomeres in the organism as the consequence of aging, in agreement with our previous findings in mice [46]. This may explain the higher mortality caused by SARS-CoV-2 infection at older ages. Indeed, we and others had previously showed that presence of short telomeres can influence the severity of COVID-19 pathologies, i.e. patients presenting more severe COVID-19 pathologies have shorter telomeres in leukocytes at different ages compared to the patients with milder disease [23, 25, 26].

These findings are also in line with previous observations showing shorter telomeres in ATII cells, but not in non-ATII cells, in fibrotic areas compared to non-fibrotic ones in PF patients [47]. Although we cannot rule out that a different outcome might have been observed in COVID-19 patients not having cancer, the fact that both cohorts, controls and COVID-19 patients, had lung cancer supports our statement that short telomeres in ATII cells associate with lung fibrosis in post-COVID-19 patients.

In conclusion, here we reveal a link between short telomere length in ATII cells and post-viral lung fibrosis outcome in post-COVID-19 patients. In particular, our results suggest a model in which the long-term maintenance of interstitial lung fibrosis in post-COVID-19 patients is triggered by short telomere length in ATII cells, which in turn could be the result of increased ATII turnover as the consequence of the viral infection, and/or pre-existing short telomeres in the patient associated to increasing age. As short telomeres can be elongated by telomerase, and telomerase activation strategies have been shown by us to have therapeutic effects in diseases associated to short telomeres, such as pulmonary fibrosis [21, 48], it is tempting to speculate that such telomerase activation therapies could improve tissue pathologies in post-COVID-19 patients such as lung fibrosis after overcoming the viral infection. Assuming at least 10% of COVID-19 survivors develop long COVID-19, and given that the global incidence reported by the World health Organization (WHO) surpasses 600 million confirmed cases at the time of writing, it is clear that long COVID-19 has become a major public health concern that needs research (https://covid19.who.int/) [49].

## MATERIALS AND METHODS

### Patient data and lung sampling

Samples and data from a retrospective cohort of patients with passed COVID-19 previously to lung surgery were obtained. Patients were recruited from the University Hospital of Marques de Valdecilla in Santander, Spain. This study presents 19 COVID-19 subjects (5 females and 14 males) and 79 controls undergoing tumor resection surgery in which tumor and some of the healthy tissue near it were taken out. Patients were matched for age and sex (35 females and 64 males of ages ranging from 42 to 84 years old) (Table 1). The biopsies were obtained near the patient’s tumor and corresponded to healthy tissue as confirmed by pathological analysis. The lung samples were paraffin-embedded.

### Histopathological and immunostaining analyses

Tissue samples were fixed in 10% buffered formalin, dehydrated, embedded in paraffin wax and sectioned at 2.5 μm. Tissue sections were deparaffinized in xylene and re-hydrated through a series of graded ethanol until water. Immunohistochemistry (IHC) were performed on de-paraffined tissue sections processed with 10 mM sodium citrate (pH 6.5) cooked under pressure for 2 min. IHC staining of Prosurfactant Protein C (SP-C) and Smooth Muscle Actin (SMA) in lung sections was performed with anti-proSPC (1:2000; Merck, AB3786) and with anti-SMA (DAKO, IR611). Histopathological analysis of paraffin-embedded lungs was performed in lung sections stained with nuclear fast red and Masson’s trichrome using standard procedures. To quantify collagen deposition Sirius red staining was performed on deparaffinised slides with picro-sirius red solution for 1 h. The slides were counterstained with hematoxylin and analyzed by light microscopy. Fiji open-source image processing software package v1.48r (http://fiji.sc) was used for the quantification of lung collagen areas.

### Immunofluorescence and quantitative fluorescence *In Situ* hybridization (Q-FISH) analysis

Lung samples were fixed in 10% formalin, paraffine-embedded and cut in 2.5-μm sections, which were mounted in superfrost plus portaobjects. Immunofluorescence was performed on deparaffinized samples processed with Tris-EDTA and cooked under pressure for 2 min for antigen retrieval. Tissues were permeabilized with 0.5% Triton X-100 in PBS for 3 h at room temperature. Samples were blocked in PBS with 4% BSA for 3h and incubated overnight at 4° C with pro-SPC antibody (1:100; Abcam ab90716). Slides were washed three times for 15 min with PBS with 0.1% Tween 20 and incubated with the secondary antibody Alexa Fluor 488 anti-rabbit (1:1000; Invitrogen A11008) for 1h at room temperature. Samples were washed three times for 15 min with PBS with 0.1% Triton X-100 and then fixed for 20 min in 4% paraformaldehyde in PBS. Quantitative FISH was performed as described before [50] with some modifications: samples were not treated with pepsin and were subjected directly to dehydration steps, formamide concentration during incubation with the probe and washes was reduced from 70% to 50% and incubation of the sample with the probe was reduced to 30 min. Telomere PNA probe labeled with CY3 (Panagene) was used. Nuclei were counterstained in a 4μg/ml DAPI/PBS solution before mounting with Vectashield (Vector Laboratories H-1000). Deparaffinized lung sections underwent antigen retrieval in 10 mM sodium citrate buffer and permeabilization was performed in PBS 0.5% Triton X-100 for 1.5 hours.

Immunofluorescence images were obtained using a confocal laser-scanning microscope (Leica TSC SP8) using a Plan Apo 63Å-1.40 NA oil immersion objective (HCX). Maximal projection of z-stack images generated using advanced fluorescence software (LAS) were analyzed with Definiens XD software package. The DAPI images were used to detect telomeric signals inside each nucleus.

## Supplementary Material

Supplementary Figure
