# Supplementary material for: Short telomeres in alveolar type II cells associate with lung fibrosis in post COVID-19 patients with cancer
Source: Aging (Albany NY). 2023 Jun 7;15(11):4625–41. doi: 10.18632/aging.204755 (PMC10292892; doi:10.18632/aging.204755)
Supplement: Supplementary Figure [file aging-15-204755-s001.pdf]

SUPPLEMENTARY FIGURE

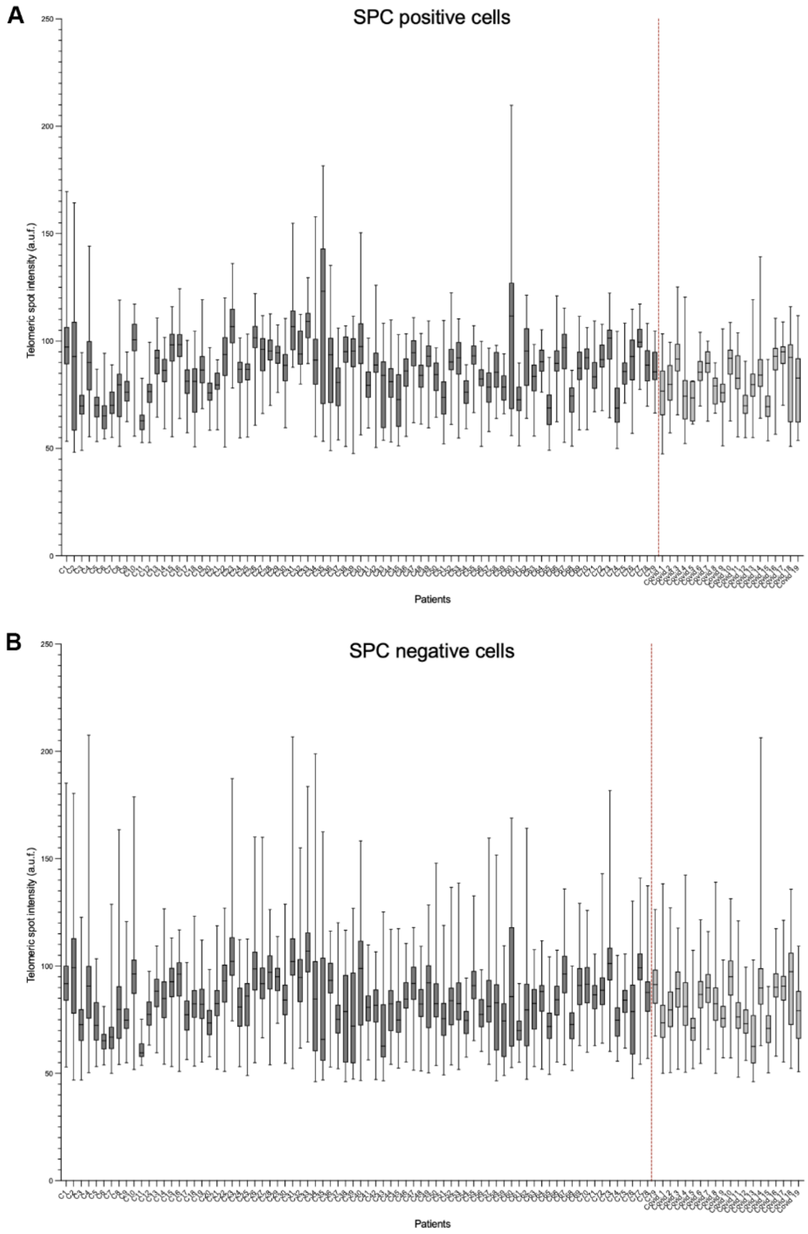

Supplementary Figure 1. Distribution of mean telomeric spot intensity per nucleus in lung tissue sections from controls and from COVID-19 patients. (**A**, **B**) Box and Whisker plot representation of mean telomeric spot intensity per nucleus in alveolar type II cells (AT2) pro-SPC positive (**A**) and in non-AT2 cells pro-SPC negative (**B**) in lung sections of control and COVID-19 patients. The ends of the box are the upper and lower quartiles so that the box spans the interquartile range. The middle line represents the median and bars the standard deviation.
